# Pregnancy after breast cancer in *BRCA1/2* mutation carriers

**DOI:** 10.1186/s13053-022-00209-1

**Published:** 2022-01-21

**Authors:** Jelena Maksimenko, Arvīds Irmejs, Jānis Gardovskis

**Affiliations:** 1 Institute of Oncology, Riga Stradiņš University, Riga, Latvia; 2 Department of Surgery, Riga Stradiņš University, Riga, Latvia; 3 Breast Unit, Department of Surgery, Pauls Stradins Clinical University Hospital, Riga, Latvia

**Keywords:** Breast cancer, *BRCA1/2* carriers, Fertility preservation

## Abstract

**Background:**

Often young women affected with *BRCA1/2* positive breast cancer have not finished or even not started their childbearing before the onset of the disease. The aim of our mini-review is to summarize state of art knowledge on pregnancy after breast cancer in *BRCA1/2* carriers.

**Methods:**

A broad review of the literature was conducted using MEDLINE (via PubMed) for relevant articles published.

**Main body of the abstract:**

This review summarizes the impact of different cytotoxic agents on a fertility, fertility preservation, maternal and fetal prognosis after pregnancy in breast cancer survivors with *BRCA1/2*.

**Conclusion:**

According to the existing literature evidence pregnancy after therapy for breast cancer in *BRCA* carriers is safe for the mother and offspring, but patients’ needs, oncofertility counseling and fertility-sparing strategy should be carefully planned before starting the cytotoxic treatment.

## Introduction

According to EUROSTAT data the average age of first-time mothers in European Union are steadily increasing over the last decade and reached the 29,3 years in 2018 [1]. Approximately, 1 in 10 women with breast cancer diagnosed under 40 years are carrying a *BRCA1 or BRCA2* mutation [2]. It means that often young women affected with *BRCA1/2* positive breast cancer have not finished or even not started their childbearing before the onset of the disease. Surprisingly, 19% of *BRCA1/2* carriers conceive within 10 years after breast cancer diagnosis [3]. However, survey study regarding expertise of specialists involved in breast care showed some gaps of knowledge regarding fertility and pregnancy management in *BRCA1/2*- related breast cancer survivors [4]. The aim of our mini-review is to summarize state of art knowledge on pregnancy after breast cancer in *BRCA1/2* carriers.

## Methods

A broad review of the literature was conducted using MEDLINE (via PubMed) for relevant articles published from 1997 up to September 2021. The search terms and strategy were developed with the help of a medical librarian specializing in systematic reviews. The search terms involved medical subject headings (MeSH). The used search terms were the following: ((((breast cancer [Text Word]) OR (breast neoplasm [Text Word])) OR (“Breast Neoplasms”[Majr])) AND ((pregnancy [Text Word]) OR (“Pregnancy”[Majr]))) AND ((“Genes, BRCA1”[Mesh]) OR (“Genes, BRCA2”[Mesh])). Ninety seven articles were identified. We included only peer-reviewed articles that considered women with *BRCA1/2* pathogenic variants and were published in English with available full-text. We also used additional search techniques, such as checking reference lists and using the Pubmed “similar articles” function of relevant publications. A total of 1540 articles were identified according to the search strategy. The titles and abstracts were screened and the full texts of potentially eligible studies were assessed. The most common reason for exclusion of studies was: 1) study population not tested or negative for *BRCA1/2* pathogenic variants; 2) grey literature: conference/congress proceedings and abstracts, book chapters, dissertations, letter to editor, opinion, case reports. Priority was given to meta-analyses, systematic reviews and multicentric studies.

Finally, we included 85 articles in the study. Only 2 out of 85 manuscripts reported cohorts on pregnancy after *BRCA1/2* positive breast cancer (248 cases in total, see Table 1). Other articles mainly addreses different aspects of cytotoxic therapy and its impact on fertility (44 out of 85) as well fertility preservation strategies 24 out of 85. In 8out of 85 and 7out of 85 articles maternal and fetal prognosis was analysed, respectively. Overlapping of topics among articles exists. All 85 articles are listed in respective chapters and references.

**Table 1:** Maternal and fetal prognosis in pregnancy after breast cancer treatment in carriers of *BRCA1/2* pathogenic variants

Reference	Number of patients	Median age at diagnosis, years	Breast cancer treatment	Median age at birth, years	Maternal prognosis	Delivery complications, %	Birth defects in fetus, %
Lambertini et al., 2020 [3, 5]	195	30	Chemotherapy in 94.4% (anthracycline and/or taxane in 96,7%); Hormone therapy in 91% (Tamoxifen and/or GnRHa in 84,7%)	35.7	No impact	11.6, no impact	1.8, no impact
Valentini et al., 2013 [6, 7]	53	32.5	Chemotherapy in 83.5%; Tamoxifen in 16.7%	35	No impact	Not evaluated	Not evaluated

### Fertility, cytotoxic therapy and *BRCA1/2* mutation

*BRCA1/2* play a critical role in the double- strand DNA break repair by homologous recombination [8, 9]. As a result of impaired DNA repair, double-strand breaks accumulate in oocytes of mice heterozygous for a *BRCA1* mutation, accelerate oocyte aging and decrease the oocyte reserve by initiating the oocyte apoptosis [10, 11]. Recent studies have shown the earlier onset of natural menopause in *BRCA1/2* pathogenic variant carriers by median 1.5–4 years compared to unaffected women [6, 12]. However, *Collins* et al., shows no difference in the age of onset of natural menopause between *BRCA1/2* pathogenic variants carriers and non-carriers [13].

According to ASCO and ESMO guidelines, the choice of the systemic neo/adjuvant chemotherapy in *BRCA1/2* carriers with breast cancer should be based on the same prognostic parameters as in the case of non-carriers [14, 15]. As 80% of *BRCA1/2* positive breast cancers are triple negative, vast majority of cases are undergoing chemotherapy [16, 17]. In the following paragraph we will report on the impact of different cytotoxic agents on a fertility.

At the moment, addition of platinum salts to anthracycline- taxane chemotherapy backbone in *BRCA1/2* pathogenic variant carriers with breast cancer remains controversial [18, 19]. According to *Byrski* et al., 61% of *BRCA1* patients, who underwent platinum-based therapy achieved pCR (complete pathological response) [20]. However, in the randomized, multicenter, phase II study a single agent cisplatinum showed a lower pCR rates in *BRCA1/2* pathogenic variant carriers with HER2 negative breast cancer compared to routine combination of doxorubicin and cyclophosphamide [21]. Exposure to doxorubicin carry an intermediate risk of losing fertility by initiating DNA double strand breaks P^− 63^- dependent apoptosis in primordial follicles as well as microvascular and stromal damage of ovaries [22, 23]. The alkylating agent cyclophosphamide carry a high risk of infertility by accelerating phosphorylation of proteins that cause primordial follicle activation with subsequent apoptosis resulting in “burnout” of ovarian reserve [24]. Cisplatin/ carboplatin cause an intermediate infertility risk by binding to DNA and triggering normal transcription and replication mechanisms [23].

Olaparib (Poly (ADP-ribose)- polymerase (PARP) inhibitor) appears promising in *BRCA1/2* pathogenic variant carriers with HER2 negative breast cancer in II-III stage. In the last randomized, double-blind, phase 3 study Olaparib significantly increased 3-year invasive disease-free survival [25]. By inhibiting ADP-ribose polymerase Olaparib blocks the repair of single-strand DNA breaks and cause the synthetic lethality in *BRCA* -deficient cells and also impairs angiogenesis by inhibiting vascular endothelial growth factor (VEGF) [26–28]. In ovarian tissue VEGF stimulates the follicular growth, promotes survival and regulates the development of primordial follicles [29]. Therefore, Olaparib has a direct gonadotoxic effect by causing genomic instability in oocytes, promoting oocyte apoptosis and indirect (antiangiogenic) effect. As result, Olaparib significantly reduces ovarian reserve and lower number of oocytes retrieved after ovarian stimulation for IVF [30]. Additionally, animal models showed an increased rate of apoptosis of *BRCA1-* deficient oocytes in response to chemotherapy-induced DNA damage compared to control [31]. These findings in the animal studies were directly in line with the clinical findings in carriers of *BRCA* pathogenic variant. Carriers of *BRCA* pathogenic variant after breast cancer treatment showed gravely diminished ovarian reserve measured using AMH levels compared to non-carriers [31–33]. In contrast, other studies show no significant difference in AMH levels at breast cancer diagnosis between carriers of *BRCA* pathogenic variants and non-carriers [34–38].

In spite of severe adverse effects of systemic therapy on a potential fertility, about 80% of carriers of *BRCA1/2* pathogenic variants, who became pregnant after therapy, conceived naturally with the median time between breast cancer and pregnancy 4.5 years. There was a longer median time between breast cancer diagnosis and pregnancy in hormone receptor-positive group compared to hormone receptor- negative group (6,3 years versus 4,0 years, respectively) [3]. This may be explained by the need for more extended hormone therapy in patients with hormone receptor positive breast cancers [39]. Carrying *BRCA1* and *BRCA2* pathogenic variant is associated with 44 and 17% of risk of developing an ovarian cancer up to the age of 80 [40]. In patients with the previous breast cancer an annual risk of subsequent ovarian cancer is 1.3% for carriers of *BRCA1* pathogenic variant and 0.8% for carriers of *BRCA2* pathogenic variants with the median age at diagnosis of 51 years in carriers of *BRCA1* pathogenic variant and 54.8 years in carriers of *BRCA2* pathogenic variant [41]. Therefore, the risk- reducing bilateral salpingoophorectomy should be considered at age 35–40 years in carriers of *BRCA1* pathogenic variant and at age 40–45 years in carriers of *BRCA2* pathogenic variant, which is another threat to a potential pregnancy [39]. However, there is still the possibility of having a full- term pregnancy in patients without simultaneous hysterectomy, if timely fertility preservation strategy is in place [42].

There are several lines of evidence suggesting the tubal origin of the ovarian cancer in carriers of *BRCA1/2* pathogenic variant [43, 44]. However, at present, prophylactic salpingectomy with delayed oophorectomy could be conducted in carriers of *BRCA1/2* pathogenic variant only during ongoing clinical trials [45–48].

### Fertility preservation for breast cancer survivors with *BRCA1/2* pathogenic variant

In general, there are two main fertility preservation strategies for patients undergoing systemic therapy. First is aimed to collect oocytes/embryo before the onset of cytotoxic therapy for a later use, while second approach is focused to the preservation of oocytes during the chemotherapy by the means of special medication.

Oocyte and embryo cryopreservation with or without previous ovarian stimulation with letrozole and follicle-stimulating hormone (first strategy) are considered to be the first-line fertility preservation approach in carriers of *BRCA* pathogenic variant before chemotherapy initiation [4, 39, 49]. Previous studies showed, that the random-start controlled ovarian stimulation is as affective as early follicular phase-start controlled ovarian stimulation with similar number of retrieved oocytes [50, 51]. However, random-start controlled ovarian stimulation still may delay the chemotherapy for 2–3 weeks [52]. So, in the cases when an urgent initiation of chemotherapy is needed retrieval of immature eggs with further in vitro maturation could be performed [52]. This procedure requires only day case laparoscopy, which is possible to fit in between disclosure of breast cancer diagnosis and start of therapy, avoiding any considerable delays. Controlled ovarian stimulation with Letrozole supplementation (COSTLES) is considered to be safe in carriers of *BRCA1/2* pathogenic variants with ER+ and ER- breast cancers, regardless of short increase in estradiol levels [53, 54].

According to the three largest randomized studies and recent meta-analysis temporary ovarian suppression with gonadothropin-releasing hormone agonists (GnRHa) during cytotoxic therapy (second strategy) is associated with reduced risk of premature ovarian failure and with possible increase in pregnancy rate with no impact on breast cancer prognosis [55–58]. 95% of early breast cancer patients aged < 40 years accepted temporary ovarian suppression with GnRHa during chemotherapy, but only 1 in 3 patients accepted oncofertility counseling and 1 in 5 patients accepted to undergo oocyte/ovarian tissue cryopreservation. Interestingly, that patients with hormone-receptor positive breast cancer accepted ovarian and/or fertility preservation more frequently compared to patients with hormone-receptor negative breast cancer. Reasons for refusal were completion of childbearing and concerns about delaying the chemotherapy [59]. Taking into account the current scientific evidence temporary ovarian suppression with a GnRHa during cytotoxic therapy should be considered as a standard option for ovarian function preservation in premenopausal breast cancer patients with or without *BRCA1/2* pathogenic variant [5]. However, in premenopausal breast cancer patients with or without *BRCA1/2* pathogenic variant temporary ovarian suppression with a GnRHa during cytotoxic therapy should not be used as the main method for fertility preservation [4, 5, 49, 60]. In patients with or without *BRCA1/2* pathogenic variant, who are planning pregnancy after breast cancer treament GnRHa should be offered only after cryopreservation procedures (if accessible) [4, 5, 49, 55, 60].

Mice heterozygous for a *BRCA1* pathogenic variants showed lower oocyte yield in response to ovarian stimulation compared to wild-type [10]. Similarly, *Oktay* et al., showed 38.5 times lower response to controlled ovarian stimulation with co-administration of letrozole with gonadotropins in *BRCA1* carriers with smaller numbers of oocytes produced compared to non-carriers [61]. Few more studies also showed premature ovarian insufficiency with decreased ovarian reserve and lower number of oocyte yield [32, 62]. In contrast, other studies showed no difference in response to IVF in *BRCA* carriers compared to non-carriers [34, 35, 63]. According to the expert meeting a possible impaired reproductive capacity in patients with *BRCA1/2* pathogenic variants should be taken into account during fertility preservation counseling [64]. The multicenter retrospective study, that included 30 centers worldwide, showed an older age and a higher delivery complication rate in breast cancer survivors harboring *BRCA1/2* pathogenic variant after assisted reproductive techniques compared to natural conception (22.7% versus 4.1%) [65]. However, according to population- based studies pregnancies achieved using assisted reproductive techniques and older age at conception were associated with higher maternal morbidity compared to natural pregnancies [66, 67].

Pathogenic mutations in the *BRCA1/2* are inherited in the autosomal dominant pattern with 50% chance of transmitting of pathogenic gene variant to each offspring [7]. In vitro fertilization (IVF) with preimplantation genetic testing (PGD) could be used to avoid the passing of copy of the mutated gene to children [68]. The meta-analysis performed by *Quinn* et al., showed that only 35% of individuals with hereditary cancer syndromes have some knowledge about PGT [69]. 33–90% of carriers of *BRCA1/2* pathogenic variant accepted the use of PGT [69]. However, carriers of *BRCA1/2* pathogenic variant with a personal history of cancer considered more likely to accept PGT compared with carriers of *BRCA1/2* pathogenic variant without personal history of cancer [70, 71].

PGT was showed as a suitable method in carriers of *BRCA1/2* pathogenic variant with and without personal history of cancer, according to the largest published experience, there 70 couples were included [72]. In addition, observational cohort study showed comparable 5-year old children physical and neurological development milestones born after PGD, IVF and natural conception [73]. However, 3-fold increase in the risk of preeclampsia was observed among pregnancies after IVF and PGT compared with pregnancies after IVF without PGT. No difference in neonatal outcomes was observed among pregnancies after IVF with and without PGT [74].

### Maternal prognosis after pregnancy and the role of carrying the *BRCA1/2* mutation

Current evidence suggests that pregnancy after *BRCA1/2* positive breast cancer treatment is safe and do not negatively influence the maternal prognosis. In the largest, multicenter retrospective study pregnancy showed no impact on distant recurrence and overall survival, regardless of hormone receptor status, in *BRCA1/2* positive breast cancer patients with the median follow-up of 8,3 years [3]. Interestingly, that in the subgroup analysis pregnancy after breast cancer treatment was associated with the improved disease-free survival in *BRCA1* carriers (144 cases) and with the possible negative impact on disease-free survival in *BRCA2* carriers. However, these results should be interpreted with caution due to the low number of *BRCA2* carriers (only 49 patients) included in the analysis. Additionally, *Valentini A* et al., showed better 15-years overall survival in *BRCA1/2* carriers (128 cases) with pregnancy-associated breasst cancer and pregnancy after breast cancer treatment compared to *BRCA1/2* carriers without pregnancy (191 cases) after breast cancer treatment (93.6 and 88.6%, respectively) [7]. In the study published by *Lambertini* et al., *BRCA1/2* carriers who got pregnant following breast cancer diagnosis were younger at diagnosis, more likely with T1 tumors, without lymphnode involvement [3]. This could be explained by the “healthy mother effect”- patients with better breast cancer prognosis decide to become pregnant more frequently [75]. It is traditionally recommended to postpone pregnancy at least 2 years after treatment of breast cancer to allow to finish adjuvant therapies and identify patients with early relapse [76, 77]. However, according to large, population- based study, 54% of breast cancer patients (62 *BRCA1/2* carriers), who became pregnant, conceived less than 2 years after diagnosis [78]. Based on oocyte maturation time it is safe for the fetus to conceive at least 3–6 months after maternal exposure to endocrine and at least 6–7 months after maternal exposure to chemotherapy or/and trastuzumab [79–81]. The most significant articles are listed in Table 1. 

### Fetal prognosis in *BRCA1/2* carriers after breast cancer treatment

Pregnancy after breast cancer treatment in *BRCA1/2* carriers does not seem to worsen fetal outcomes. Delivery complications was detected in 11,6% and was similar to observed in the general population [3]. According to *Kwiatkowski* et al., *BRCA1/2* carriers has for 36% fewer miscarriages compared to non-carriers [82]. Several other studies also have reported that prior exposure to anticancer therapy did not increase the risk of congenital anomalies and miscarriages in *BRCA1/2* positive breast cancer survivors compared to the general population (1.8 and 10.3% versus 2.3–3 and 13.5%, respectively) [83–85]. However, there is an increased risk of congenital multimalformations in offsprings from families known to carry the *BRCA* mutation [86]. *BRCA-* deficient embryos have defective double-strand DNA breaks repair by homologous recombination as a result early mutations accumulates causing malformations [86, 87]. The most significant articles are listed in Table 1.

## Conclusion

Only two studies with limited number of cases have been published on the subject. According to the existing literature evidence pregnancy after therapy for breast cancer in *BRCA* carriers is safe for the mother and offspring, but patients’ needs, oncofertility counseling and fertility-sparing strategy should be carefully planned before starting the cytotoxic treatment. Further studies are necessary to strengthen the body of evidence.

## Data Availability

Not applicable.
